# Optimal Control of a Reverse Osmosis Plant for Brackish Water Desalination Driven by Intermittent Wind Power

**DOI:** 10.3390/membranes12040375

**Published:** 2022-03-30

**Authors:** Emad Ali

**Affiliations:** Chemical Engineering Department, King Saud University, Riyadh 11421, Saudi Arabia; amkamal@ksu.edu.sa

**Keywords:** water desalination, reverse osmosis, wind power, control, optimal

## Abstract

This work addresses the design of an online control system that continuously regulates a reverse osmosis (RO) desalination plant driven by wind power aided by water storage tanks. The control objective is to produce the exact hourly water demand in the presence of wind power intermittency, disturbances, and operational limitations. The manipulated variables are the RO feed pressure and the active number of RO vessels. The control system helped to decrease the annual water deficit by 20% under nominal conditions and when the plant is under the influence of disturbances. Moreover, the control system managed to decrease the annual water deficit by 73% when the plant operated under a shortage of an active number of wind turbines and RO vessels. The loss of redistributed production ratio (*LPb*) and the loss of raw production ratio (*LP*) were used as the controlled variables representing the proposed control objective. *LPb* was superior to *LP* by creating conservative control actions that produce the required water demand without violating the required water purity.

## 1. Introduction

Undoubtedly, water is an indispensable source of human life. Humans consume water in every aspect of life. As the population and their daily needs around the globe are intensifying, a steady supply of freshwater becomes very demanding. For a long time, the principal route to supply potable water was desalinating brackish water [1] and seawater [2]. Currently, around 38 × 10^9^ m^3^/day of desalinated water is produced from more than 18,000 desalination plants around the world. It is expected to increase to 38 × 10^9^ m^3^/day by 2030^3^. At the same time, the cost of and request for energy are surging, and the environmental restriction on fossil fuels is becoming more stringent. In fact, the energy consumption of desalination plants is estimated to be around 75.2 × 10^6^ Wh every year [3]. This gives impetus to researchers and practitioners to investigate energy-efficient desalination technology and/or integrate the existing desalination technologies with environmentally friendly and the least expensive energy resources. Desalination by reverse osmosis (RO) membranes is known for being the most energy-efficient technology. According to Dashtpour and Al-zubaidy [4], the specific energy consumption of seawater desalination by the RO process is 3 to 10 kW/m^3^. Most of the energy is exhausted as electricity by a high-pressure pump to feed saline water to the membrane. It is reported that a 25% fluctuation in electricity costs leads to an 11% alteration in the water cost per cubic meter [5]. Due to the expanding and fluctuation energy cost and the harmful impact of fossil fuel on the environment, it became imperative to run desalination plants employing renewable energy sources (RES). This situation was the main driver for immense research activities investigating the integration of RO desalination systems with solar or wind energy. 

Because Saudi Arabia is an arid country, it relies heavily on water desalination. Besides, because Saudi Arabia is developing rapidly, the demand for pure water and electric energy is surging. For this reason, the country set a plan to subsidize the conventionally generated electricity by RES. The nation’s 2030 vision is targeting 40 GW of solar energy and 17 GW of wind energy [6]. In fact, the country possesses a promising source of wind energy. Wind speeds greater than 3.5 m/s are observed over wide areas of the nation [6]. Moreover, wind energy is well known for being harmless to the environment and inexpensive compared to the other fuel-based energy sources [7]. 

The literature contains an abundance of efforts investigating the incorporation of wind power to drive RO desalination plants [8,9]. The focus of these efforts is to study the feasibility of operating the desalination plant smoothly during fluctuating and discontinuous wind energy. In fact, wind speed intermittency poses the main challenge against the application of wind energy to power RO plants. For example, Park et al. [10] investigated the ability of wind to supply the RO system with the required load without relying on batteries necessary to offset the power losses. They found that the best way to overcome the power discontinuity is to implement well-designed control systems. Carta et al. [11] carried out an experimental study on using wind farms to power several desalination plants including RO systems. This study focused on stand-alone plants that are disconnected from the electricity network and energy storage. They concluded that the reverse osmosis method is the most suitable for integration with wind power in several desalination plants with fixed pressure and flow rate. Charrouf et al. [12] studied the use of hybrid solar and wind energy sources supported by energy buffering to operate an RO desalination plant. The process can operate smoothly by adapting a power management protocol using artificial neural networks. Similarly, Peng et al. [13] addressed the effectiveness of a coupled system consisting of a wind turbine, a photovoltaic panel, and a battery bank to power a reverse osmosis system. They used state-of-the art optimization tools to optimize the size of the overall system while maintaining the continuity of load supply under wind variation. Cabrera et al. [14] also investigated the use of neural networks to analyze the operation of a seawater RO plant using intermittent wind energy as its main source of power. They studied the system under two operation modes. One mode was based on constant pressure and flow rate supported by energy storage and the other on variable pressure and flow rate without energy storage. Carta et al. [15] conducted a study on the use of wind-generated energy to operate a small-scale prototype seawater reverse osmosis (SWRO) system. To ensure a continuous supply of energy to the desalination process, a supercapacitor bank was utilized. Similarly, Richards et al. [16] used a supercapacitor energy bank to subsidize load intermittency due to real wind fluctuations applied to a brackish water RO system (BWRO). Lai et al. [7] outlined different policies to mitigate the harmful influence of heavily variable wind energy on the operation of RO desalination plants. They classified the solutions into three main approaches: using a battery, using hybrid RES, and adapting the system operating conditions. Mohamed and Papadakis [17] performed techno-economics of an RO desalination plant operated by coupled solar and wind energies assisted by batteries. It is reported that economics can be improved by 48% by reducing energy consumption by incorporating pressure energy recovery devices. Khiari et al. [18] also considered the use of hybrid solar–wind energy sources for an autonomous BWRO system. It was concluded that the implementation of an online control system is necessary to maintain a steady electrical load. Furthermore, they emphasized the important role of energy management policies to schedule and link the RES to the power line. Additional information about the joint solar-wind energies and their implementation in water desalination by RO systems can be found in recent review papers [19,20]. Recent work on the intermittent operation of RO is reported. For example, Garcia and Nuez [21] studied the effect of long-term intermittency on an existing brackish water RO plant. They reported declines in the permeability and increases in the energy consumption and concluded that care should be considered when designing RO plants powered by renewable energy. Elmaadawy et al. [22] compared two scenarios of hybrid renewable energy systems for RO desalination plants. In addition, the size of the best scenario was optimized to achieve economic and environmental standards. Carta and Cabrera [23] proposed a control strategy based on a genetic algorithm to optimize the operation of a seawater RO plant driven by wind power. They pointed out that the proposed strategy operates more economically than the standard system. Wang et al. [24] investigated the use of a real-time optimization-based control approach to operate a desalination plant under time-varying wind energy. They concluded that the proposed strategy can promote the use of renewable energy for RO desalination. Garcia and Nuez [25] also studied the performance of an RO system under variable operating conditions. They suggested care should be made in selecting the appropriate membrane type in terms of the permeability coefficient. Dimitriou et al. [26] developed a dynamic model for RO to study the physical compaction due to variable operating conditions. They observed a drop in the mass flux when the operating pressure suddenly changed. In summary, wind speed intermittency poses difficulty in designing and operating an RO system smoothly. The coupled RES systems are even more complex because they incur extra instrumentation and control elements [18]. Furthermore, hybrid systems are more expensive as the capital cost of photovoltaic panels is relatively higher [17]. 

Ali et al. [27] conducted a comprehensive optimal design of an RO desalination plant powered by wind power. The grassroot design considered using storage tanks or energy storage devices to ensure smooth operation during wind power fluctuation. The study pointed out that the use of storage tanks is less expensive and complicated than that using batteries. This grassroots design and similarly other reported ones focus on how to improve the plant performance during wind fluctuation but do not consider other types of disturbances. Using the design approach that optimizes the power generation system to deliver a constant load to the RO plant can guarantee smooth operation. Since the load supply is steady, the feed pressure and associated feed flow rate will be steady as well. This design approach may not produce the exact instantaneous production rate. For example, when the available renewable energy sources are zero and the battery is empty, a loss of production will take place. Cabrera et al. [14] concluded that working with a steady mode led to less continuous operation than the variable pressure and flow rate. Moreover, when the RO process undergoes sudden disturbances such as fouling, maintenance, and variation in the feed salinity, the production rate and/or product quality may degrade. Since no automatic control is involved, no corrective actions will be taken. Even if a control system exists, the spectrum of variation in the feed pressure/flow rate is limited because the supplied power is fixed. On the other hand, the design approach that optimizes the production rate by storing excess water for later uses may lead to a better realistic operation. For example, when the power supply is zero, the RO plant will stop but the production line will be maintained by offsetting the deficit by the stored water. Moreover, since an RO plant operates under a variable supplied load, it will incorporate automatic control to adapt the feed pressure and flow rate to the variable available power. This feature will foster the plant operation in the presence of unforeseen turbulence. Park et al. [10] also recommended using control strategies to counter the intermittent operation especially when the feed water is highly concentrated. Hence, the objective of this work is to design an appropriate online control objective to run the plant smoothly during wind intermittency. The effectiveness of the proposed control system to reject the effect of disturbances overlooked by the grassroots design will also be assessed. The novelty of the work centers around varying the feed pressure and the number of active vessels by an external control system not freely by the wind fluctuation. To the author’s knowledge, an online adaptation of the active number of vessels has not been considered. 

## 2. Description of the Desalination Plant

**Figure 1:**
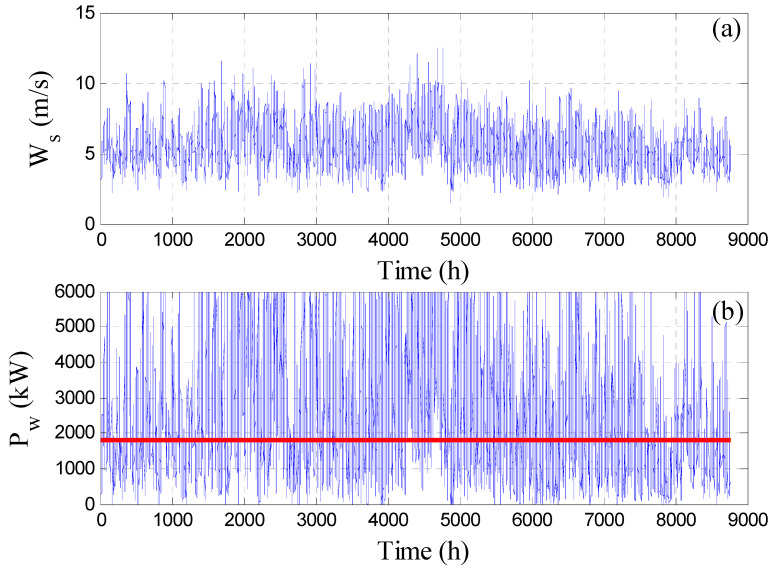
Annual wind property profile for the site (**a**) wind speed, (**b**) generated power.

Here, we consider a large capacity RO pant plant driven by wind energy. The plant is designed to produce 2592 m^3^/h of freshwater that requires 1780 kW of wind power each hour [28]. The RO process is used to desalinate brackish water of salinity of 1 kg/m^3^. The topology of the plant is given in [28] and consists of a large number of RO vessels arranged in parallel and powered by a single power line. The power line obtains its energy from a large number of turbines that harness the available wind power of the designated site. According to the previous optimal grassroots design of the plant [27], 100 wind turbines, 701 RO vessels, and storage tanks of 1 × 10^6^ m^3^ capacity are required to operate the plant at nominal capacity. The well-designed plant can provide a smooth production rate even in the presence of wind power intermittency by storing surplus water produced during high wind power periods and use it to compensate for water losses during low wind power periods. Hence, in this study, a stand-alone wind-driven RO plant without a battery is considered. The available wind speed and its associated wind power generated from 100 turbines are shown in Figure 1 using a typical wind power model [28,29]. The profile of the wind power shown in Figure 1b will be utilized in the entire simulation presented in this study. The expression of the mathematical model governing the RO process was developed previously [28,30] and is listed here in Appendix A. More details about the overall plant, the wind power system, the RO process, the values of the physical and design parameters of the overall plant, and the method of solution of the RO model can be found in previous work [27,28]. 

## 3. Water Storage Mechanism

Due to wind power fluctuation, the hourly (instantaneous) water production varies significantly. In this case, the hourly loss of water production can be defined as:

(1) $$LP\left(t_{k}\right)=Q_{p}^{s}\left(t_{k}\right)-Q_{p}\left(t_{k}\right)$$

*LP* measures the difference between the desired hourly production ($Q_{p}^{s}$) and the actual hourly production ($Q_{p}$). Hence, positive *LP* indicate a loss of hourly production, i.e., the desired production cannot be met, zero *LP* means the exact desired hourly production is met, and negative *LP* means surplus water beyond the target is produced. In this case, the surplus of water produced each hour beyond the hourly demand, i.e., when *LP* < 0, is transferred to storage tanks for later use. Whenever the hourly produced water is less than the hourly demand, i.e., *LP* > 0, the supplied water is compensated by withdrawing the difference from the storage tanks. Therefore, the hourly stored capacity can be computed at any sampling hour (*t_k_*) as follows:

(2) $$TS\left(t_{k}\right)=TS\left(t_{k-1}\right)-LP\left(t_{k}\right)$$

Subject to the following constraint:

(3) $$TS\left(t_{k}\right)\geq 0$$

*TS* measures the amount of water accumulated in the storage tank each hour. During deficit production, i.e., positive *LP*, the deficit is withdrawn from the storage tank. During surplus production, i.e., negative LP, the surplus water is added to the tank. The above is applied for *k* = 1 to 24 hours comprising one day of operation. Note that no upper limit on the storage capacity is imposed because no excess permeate shall be rejected. The hourly production will be distributed according to Equation (1). This means that at each hour when water is produced in excess, the excess will be stored in the tanks, while when water production is limited, water is withdrawn from the tanks and added to the delivery line to compensate for the losses. The redistributed production rate will be denoted here as the flow rate in the supply pipelines. Both terms will be used interchangeably throughout the manuscript. Thereby, the hourly production and the hourly loss of production in the pipelines, i.e., after redistribution of the produced water, are defined as follows:

(4) $$Q_{p}^{r}\left(t_{k}\right)=Q_{p}\left(t_{k}\right)-\left(TS\left(t_{k-1}\right)-TS\left(t_{k}\right)\right)$$

(5) $$LPb\left(t_{k}\right)=LP\left(t_{k}\right)-\left(TS\left(t_{k-1}\right)-TS\left(t_{k}\right)\right)$$

$Q_{p}^{r}$ quantifies the hourly water production in the pipelines, i.e., the water taken from the plant and pumped towards the municipal station each hour. It comprises the actual hourly amount produced from the plant plus the amount taken from the storage tank during the deficit or minus the amount sent to the tank during surplus. The ideal case is that $Q_{p}^{r}$ equals $Q_{p}^{s}$. Consequently, *LPb* is the state of the hourly loss of production in the pipelines.

## 4. Control Objective Formulation

Most grassroots design procedures determine the total required turbines, vessels, and other associated components such as storage tanks and/or energy storage banks. Usually, this design approach is based on minimizing a specific cost index such as the water cost. Although the design approach is meant to withstand dynamic wind speed fluctuations, it is based on the proposition of steady operating conditions. Moreover, the cost index is usually a lumped value of a time-varying output over a long period. Hence, the instantaneous variation of the key output is not considered explicitly in the cost index. In a real application, operating conditions may divert from the nominal case. For example, feed salinity may change. The RO performance may degrade due to fouling and scaling. Furthermore, a portion of the RO vessels and/or turbines may undergo shut down for maintenance. All of these situations create disturbances in the plant operation. In this case, the implementation of control systems is indispensable to regulate the RO operation at each sampling instant in the face of possible disturbances. For the given plant, the feed pressure of the RO vessel is a crucial manipulated variable. The feed flow rate of the RO cannot be manipulated because it is dependent on the feed pressure for given supplied power. Nevertheless, the active set of operating vessels can be adjusted online, which will regulate the delivered power per vessel. The manipulated power per vessel will indirectly regulate the feed flow rate. Therefore, *P_f_* and *N_v_* are candidate manipulated variables for the control system. 

For RO operation, it is necessary to control the production rate and permeate quality. Usually, controlling the permeate quality exclusively sacrifices the production rate, but by controlling the production rate solely, the permeate salinity can be maintained except under certain circumstances. It is difficult to have more than one control objective because this will lead to the use of a weighting factor to compromise between competing objectives. Similarly, it is difficult to incorporate secondary objectives as a hard constraint because this will also lead to infeasible solutions. Therefor, it is prudent to select the most appropriate control objective function. Besides, it is instructive to investigate the different possible control objectives along with their weakness and benefits. 

When dealing with the production rate as a control objective, there are three options. The overall production, i.e., total production over a specific interval, instantaneous production, or distributed production. It is interesting to study the plant performance using the different options for the control objective in terms of the production rate. The designated control system can be applied under two different approaches: the optimal control approach which computes the future input trajectories in an open-loop mode fashion and the typical feedback control approach which computes the instantaneous corrective input each sampling instant [31]. Both approaches will be studied. 

The optimal control strategy can be formulated as numerically solving the following optimization problem:

(6) $$\underset{P_{f}\left(t_{k}\right)\cdots P_{f}\left(t_{N}\right),N_{v}\left(t_{k}\right)\cdots N_{v}\left(t_{N}\right)}{\min}\varphi$$

Subject to:

(7) $$3\leq P_{f}\left(t_{k}\right)\leq 50;\;k=1,\cdots ,N$$

(8) $$10\leq N_{v}\left(t_{k}\right)\leq 700;\;k=1,\cdots ,N$$

(9) $$2.5\leq Q_{f}\left(t_{k}\right)\leq 15;\;k=1,\cdots ,N$$

(10) $$C_{p}\left(t_{k}\right)\leq 0.4;\;k=1,\cdots ,N$$

In the optimal control algorithms, the effect of internal disturbances is not compensated for via the feedback system. This control system manipulates simultaneously a set of values for *P_f_* and *N_v_* over a specific period to maintain a certain control objective (φ) formulated as a single lumped value. The desired control objectives φ can be written in the following forms as follows:

Loss of total production ratio:

(11) $$\varphi \equiv LPTR=\frac{\sum_{k=1}^{k=24}LP\left(t_{k}\right)}{\sum_{k=1}^{k=24}Q_{p}^{s}}$$

Loss of total hourly production ratio:

(12) $$\varphi \equiv LPTHR=\frac{\sum_{k=1}^{24}\left|LP\left(t_{k}\right)\right|}{\sum_{k=1}^{24}Q_{P}^{s}\left(t_{k}\right)}$$

Loss of total hourly redistributed production ratio:

(13) $$\varphi \equiv LPTHbR=\frac{\sum_{k=1}^{24}\left|LPb\left(t_{k}\right)\right|}{\sum_{k=1}^{24}Q_{P}^{s}\left(t_{k}\right)}$$

*LPTR* is the total loss of production over one day divided by the total desired production over one day. Note that *LPTR* can be positive if the surplus water exceeds the shortage over a period of one day and vice versa. In this case, the control strategy forces the average value of *LP* over one day to zero. *LPTHR* is the sum of absolute values of LP over one day divided by the total desired production per day. Since *LPTHR* is always positive, the control strategy will force the hourly value of LP to zero. *LPTHbR* is the same as *LPTHR* but applies to the production in the pipelines. The above objective functions are estimated by solving the RO process given in Appendix A. Note that by using LPTR as an objective function, the optimization will force *LPTR* to a negative value to maximize the total surplus production of water. This will increase the opportunity of offsetting any hourly water deficit during the day. However, it may produce additional un-necessary water production. When *LPTHR* is used, the controller will force the value of *LPTHR* to zero that is making the RO process produce the exact hourly demand over the entire period of one day. This may not be achieved because of the variation of the supplied wind power. For *LPTHbR*, the optimization will force *LPTHbR* to zero. In this case, the controller will reduce un-necessary surplus water production by trying to produce an excess of water sufficient to compensate for the water shortages during a day of operation. It should be noted though, regardless of the used control objective, the ultimate goal of the control system is to achieve a zero value for *LPTHbR*. The superiority of the candidate objective function is not straightforward because of the physical constraints such as the upper and lower limits on the feed flow rate and the active number of vessels. Moreover, the permeate salinity must be lower than the acceptable limit for drinkable water.

For the instantaneous (feedback) control system, the control algorithm can be formulated as follows:

(14) $$\underset{P_{f}\left(t_{k}\right),N_{v}\left(t_{k}\right)}{\min}\varphi \left(t_{k}\right)$$

Subject to:

(15) $$3\leq P_{f}\left(t_{k}\right)\leq 50$$

(16) $$10\leq N_{v}\left(t_{k}\right)\leq 700$$

(17) $$2.5\leq Q_{f}\left(t_{k}\right)\leq 15$$

(18) $$C_{p}\left(t_{k}\right)\leq 0.4$$

The instantaneous control system is conducted every sampling instant to compute the current value of *P_f_* and *N_v_* to achieve the instant desired objective function, $\varphi \left(t_{k}\right).$ The objective function in this case is also a time-varying parameter. In this study, *LP* and *LPb* will be used as an objective function, which will be also discussed further in the following section. It should be emphasized here that the goal is not developing a new control algorithm nor advancing the existing control algorithms or theory. The aim is to assess the importance and effectiveness of using online control systems to regulate a desalination plant in the face of unanticipated disturbances. Another goal is to present an additional tool to adapt the plant operation during severe wind speed fluctuations. Moreover, the control algorithms are not formulated in the standard control forms. The control algorithms are expressed as an optimization problem for simplification. First, the above formulation does not require careful adjustment of the controller settings found in typical control systems. Second, the above formulation allows incorporating physical constraints in an optimal fashion. The constraint on *C_p_* is not enforced unless stated. 

## 5. Discussion of Results

**Figure 2:**
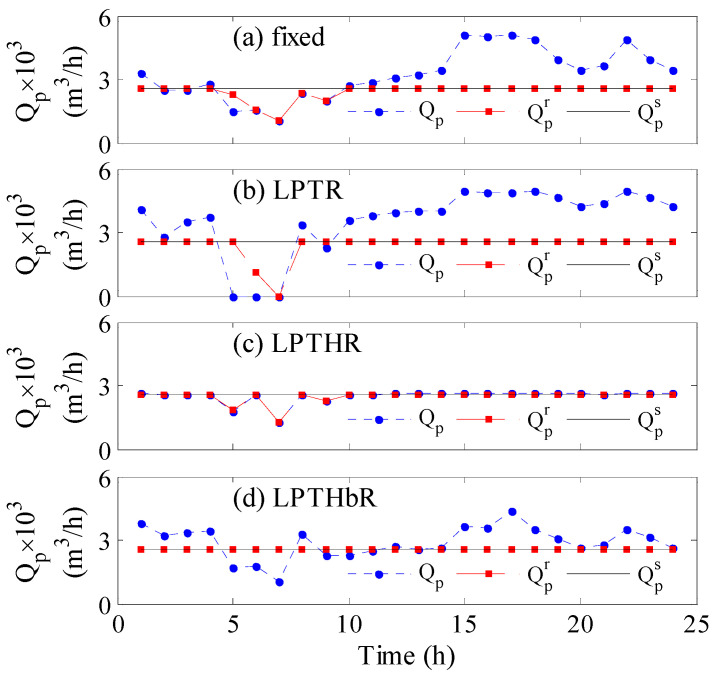
Time response of the production rate; (**a**) no control, (**b**–**d**) using optimal control with different objective functions.

**Figure 3:**
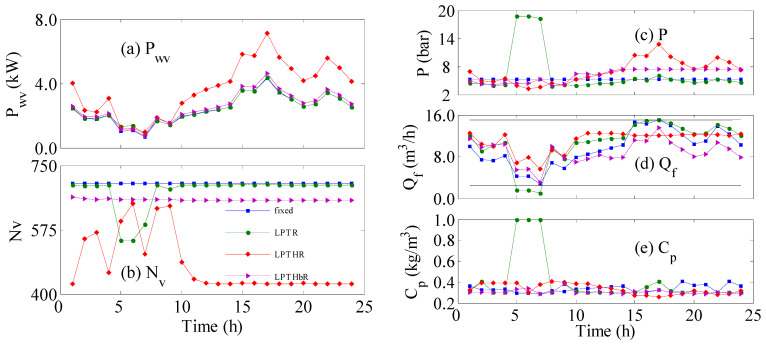
Variation of the rocess parameter with time, (square): fixed; (circle): *LPTR*; (diamond): *LPTHR*; (triangle): *LPTHbR*.

Figure 2 illustrates the evolution of the production rate with time for different controlled parameters. Specifically, Figure 2a shows the results when no control strategy is employed while Figure 2b–d depict the outcome when the control strategy is active using different control objectives as indicated inside the individual figure. The time response covers a 24-h operation assuming the storage tank is empty initially. Figure 2a demonstrates the actual hourly production rate (dot line), the hourly redistributed production (dashed line), and the target hourly production (solid line) when the feed pressure and the number of active RO vessels are constant at the nominal value obtained by the grassroots design. The actual hourly production is low in the first 10 hours and increases afterward, following the trend of the available wind power shown in Figure 3a. Since the wind power is low especially between 5 and 10 h, the resulting actual hourly production rate becomes lower than the target value. Hence, the actual production and consequently the redistributed production suffer from shortages in the interval of 5 to 10 h of operation. The drop in the production rate is due to the drop in the wind power at that period as shown in Figure 3a. The decline in the redistributed production, which amounts to 5.73% (Table 1, first column), is ascribed to the fact that the storage tanks are not sufficiently filled to compensate for the losses. The insufficient filling of the tanks is because the actual production before 5 h was not in a large excess. This deficiency can be treated by initial filling of the storage tank or via continuous operation. However, this option will not be considered for demonstration purposes and comparison with the outcome of the other control objectives. 

When no control strategy is employed (Figure 2a), the production in the pipeline ($Q_{p}^{r}$) does not meet the municipal requirement during the 5 to 10 h. Hence, the proposed control system is employed to overcome the shortage as shown in Figure 2b–d. Figure 2b depicts the results when the LPTR is used as the objective function in the control strategy, which shows how the overall production intensifies, manifested by increased production in the early 5 h and the final 14 hours, leading to a 38% water surplus (Table 1). However, since the LPTR control objective is an average value, the control system ignored the zero production in the 5–7-h period because it is submerged in the total value of the production. The zero production causes the largest shortage in the redistributed production among all other cases. When the controller targets the redistributed production rate (Figure 2d), perfect tracking of the desired production rate is obtained. Although both LPTHbR and LPTHR are lumped values, unlike LPTR, they consider the instantaneous value instead of the overall value. However, LPTHR (Figure 2c) is found inferior to LPTHbR in terms of sacrificing the water supply, i.e., the total loss of production in the pipelines is 4% compared to 0. The performance of the control objectives shown in Figure 2 can be explained by the response of the corresponding manipulated variables shown in Figure 3. 

Figure 3 illustrates the temporal behaviour of various process parameters that correspond to the results and cases shown in Figure 2. Figure 3a depicts the supplied wind power per vessel, which is simply the available wind power divided by the number of active vessels shown in Figure 3b. The selected 24-h portion of the wind power undergoes apparent variation but does not reach zero values. The *P_wv_* for all cases is almost similar in magnitude and trend because they have an almost constant value of *N_v_* (Figure 3b) except for *LPTR* and *LPTHR*. In the early 10 h of operation, *LPTHR* commanded some continuous alteration in *N_v_* and subsequently altered P_wv_. At time longer than 10 h, LPTHR predicted a constant *N_v_* but much lower than that of the other cases. This made the corresponding *P_wv_* higher than in the other cases. Similarly, *LPTR* exhibited sudden variation in *N_v_* between 5 and 10 hours of operation. Because the sudden changes were small, its reflection on *P_wv_* was also minor and moreover was obscured by the overlapping curves. Figure 3c illustrates how the controller adapts the feed pressure to achieve the desired control objective. For *LPTR*, the controller increases *P_f_* considerably during the 5~10-h period to increase the production because the wind power during that period is low. However, this action reduces the feed flow rate to below the minimum value (Figure 3d), which disables the RO vessels, causing zero production and zero salt removal, i.e., *C_p_* = 1. Since Q_f_ is not involved in the control objective, i.e., not used as design parameter because it is not independent variable, the controller cannot make corrective actions. Consequently, a total of 5.6% of losses in the pipeline’s flow is observed. For *LPTHR*, the controller generated initial aggressive actions in *N_v_* (Figure 3b) in order to intensify the delivered power per vessel during low wind speed. In addition, the controller raised the feed pressure after 10 h of operation (Figure 3c) to make use of the surplus supplied power. The controller made these actions because it tries to make the raw production exactly equal to the desired demand without relying on excess water production. This is evident in Figure 2c as the raw production rate is identical to the redistributed production rate. This control objective suffers from a 4% deficit because it does not produce excess water. *LPTHbR*, on the other hand, delivered the best performance with zero losses in the pipeline flow. Although the controller in this case commended a steady value for *N_v_* over the 24 h, it was fixed at a lower value than that of the nominal case (fixed; i.e., no controller). Moreover, the controller delivered a balanced and smooth variation of the feed pressure over the day. We can argue that *LPTHbR* is the best control objective. However, the above approach (optimal control) cannot be implemented easily because it requires the knowledge of future wind power fluctuation. Model predictive control (MPC) [32] uses a prediction horizon to extrapolate the process prediction into the future. However, MPC will assume the future inputs and disturbance are fixed at the current value. Therefore, we will investigate a control system that does not acquire future predictions such as the instantaneous control algorithm.

The instantaneous control system is typically implemented each sampling time to correct the manipulated variable to achieve a specific control objective. This type of control system can be implemented in a closed-loop fashion to compensate for the effect of unknown disturbances. Testing this control approach for two control objectives, namely, *LP* and *LPb* (Equations (1) and (5)), gives the results in Table 2. Since this control algorithm is implemented in each sampling instant, the lumped control objectives, i.e., *LPTR*, *LPTHR*, *LPTHbR*, are meaningless. For both control objectives (*LP* and *LPb*), we tested the control performance with and without the inclusion of an upper bound on *C_p_* to guarantee acceptable permeate quality. 

According to Table 2, LP without disturbance (nominal) maintained excellent hourly redistributed water production manifested by *LPTHbR* equal to zero but with high average water salinity (*C_p_* = 0.485 kg/m^3^). The situation improved in terms of the average water purity by incorporating an upper constraint on *C_p_* without sacrificing the production. In this case, *C_p_* was reduced to 0.414 kg/m^3^ while *LPTHbR* remained zero.

**Figure 4:**
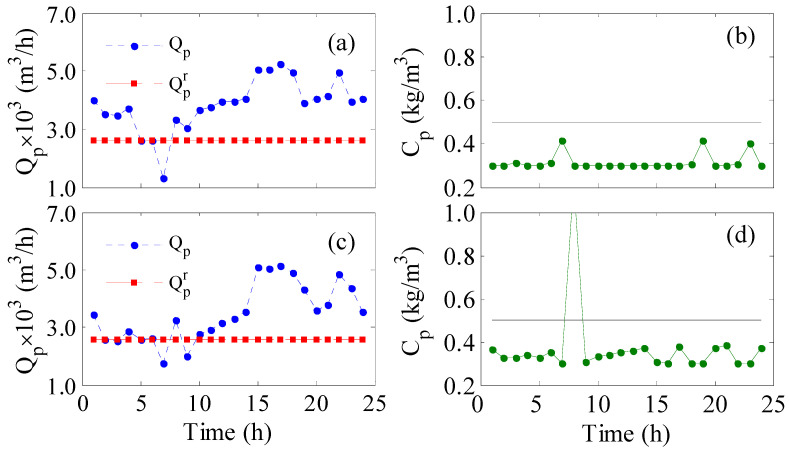
Instantaneous control results for bounded *LP* (**a**,**b**) and unbounded *LPb* (**c**,**d**).

For unbounded *LPb*, the performance was excellent in the sense of maintaining 100% satisfaction of the desired production (*LPTHbR* = 0) and average product quality (*C_p_* = 0.363). In fact, the obtained *C_p_* was superior to that for *LP* case. When the constraint on *C_p_* was enforced, the redistributed production rate was still acceptable. However, the raw hourly production was slightly degraded as the shortage increased from 19% to 29% as manifested by *LPTHR*. Therefore, plant performance may degrade under more severe conditions. Comparing *LP* with *LPb* objectives, *LP* tended to increase the water surplus manifested by a larger value for LPTR; however, this was at the expense of higher permeate salinity. Furthermore, the unbounded *LP* led to the highest permeate salinity. Furthermore, as the values of *LPRT* in Table 2 indicate, the process must produce at least a 33% surplus of water in order to achieve 100% satisfaction of the hourly water demand. It should be noted that regardless of whether the control system incorporates a constraint on *C_p_* or not, the instantaneous permeates salinity may violate the upper bound as shown in Figure 4. At specific sampling instants where the transmembrane pressure becomes low relative to the feed flow rate, the mass transfer of the solute is hindered, leading to poor permeate purity. Nevertheless, the hourly produced freshwater is mixed together in the storage tank diluting these poor products. Hence, the use of average water salinity is an acceptable metric. Note the dynamic response of the other parameters, e.g., feed pressure feed flow rate, etc., are excluded from Figure 4 to reduce the number of illustrations. Nevertheless, incorporation of the control system improves the plant performance over that of the uncontrolled system shown in Figure 2a in terms of 100% fulfillment of the hourly production.

**Figure 5:**
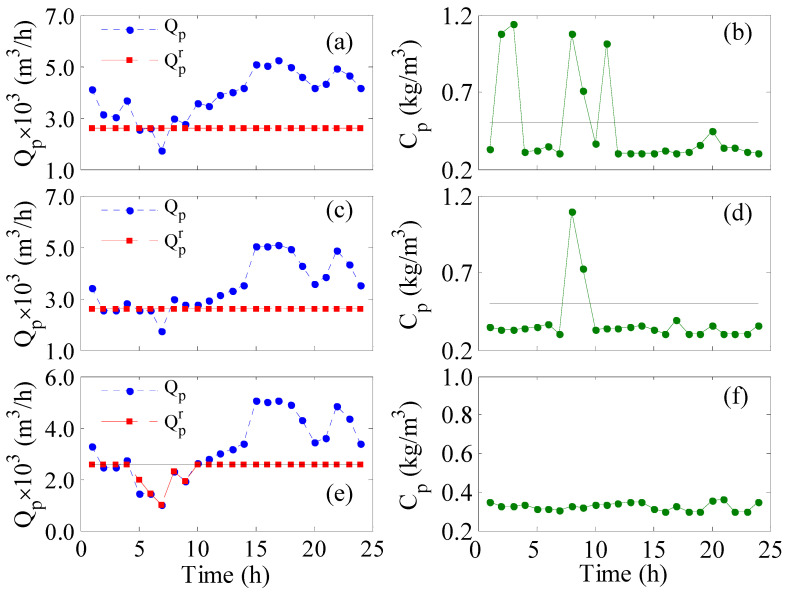
Instantaneous control results in the presence of disturbances (**a**,**b**): Bounded *LP*; (**c**,**d**): unbounded using *LPb* objective; (**e**,**f**) fixed.

We further tested the instantaneous control system when the plant underwent unforeseen disturbances other than the intermittent wind power. For this case, we assumed that feed salinity increased by 50% to reach 1.5 kg/m^3^ and that the membrane permeability decreased by 20%. The latter represents mass transfer degradation due to fouling. The performance of the fixed (uncontrolled) and controlled RO plant is listed in Table 2 and depicted in Figure 5. According to Table 2 and Figure 5e,f, the disturbance influenced the operation when no control strategy was used and showed a 7% loss in the pipeline production (*LPTHbR*). Obviously, the controlled plant managed to maintain the desired redistributed production for both control objectives (*LP* & *LPb*) as shown in Figure 5b–c. This is because the control system leveraged the high wind periods to produce surplus water. However, permeate purity was affected, especially in the unbounded *LP* case, which reached an unacceptable value of about 0.6 kg/m^3^ (Table 2). Figure 5b,d show spikes in the permeate salinity as specific instants. This is because the controller lowered the feed pressure to accommodate more feed flow, which sacrificed the permeate purity. This means the transmembrane pressure was not sufficient to separate the salt from the solution to a larger extent. Park et al. [10] have also pointed out that the balance between productivity and water quality is challenging and that high average wind power is needed to operate the system within safe operating conditions. On the other hand, the production rate in the uncontrolled case suffered from additional losses whereas the deficit increased from 5.73% in the nominal case (Table 1) to 7% in Table 2. Nevertheless, the permeate purity was unaffected as shown in Figure 5f. This can be considered an advantage of the uncontrolled system as the feed pressure was kept constant at an optimal value that did not sacrifice the water purity during feed flow fluctuation.

**Figure 6:**
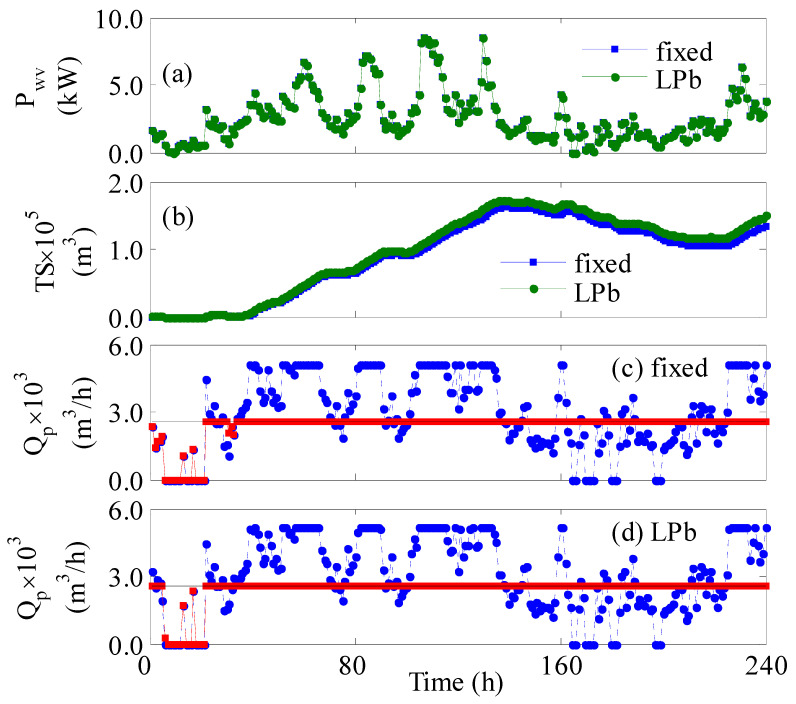
Plant dynamic response for 10 days under nominal conditions; (**a**,**b**) square marker: fixed, circle marker: *LPb*; (**c**,**d**) solid line: $Q_{p}^{s}$; blue circle: $Q_{p}$; red square: $Q_{p}^{r}$.

The process was further assessed under a shortage of the existing number of RO vessels and turbines. The process was simulated assuming 20% of the turbines and 200 vessels are laid off for regular maintenance. Since the optimal design of the plant requires the full availability of turbines and vessels, the performance may degrade. Thereby, this would present a good test for the online control system to overcome such situations. Before discussing the effect of the partial operation of the plant, some aspects of the nominal operation will be highlighted first. The nominal operation, i.e., without shortages in the units, over 10 days is shown in Figure 6. The figure compares the performance of the uncontrolled and controlled process. Figure 6c,d illustrate a clear water deficit in the first 30 hours of operation regardless of employing a controller or not. If the null/minimum wind power occurs (Figure 6a) in the first-time operation where no previous surplus water being stored (Figure 6b), water deficit in the pipelines is inevitable. When the zero or very low wind power occurs later in the period of 160 to 220 h, the process cannot produce new water, but the overall production is not affected due to the presence of previously stored water. Figure 6b shows an abundance of water is stored prior to the operation time of 160 h. In general, when the wind power is low, the control system can still provide improvement by lowering the feed pressure and/or the number of the active set of vessels. Lowering the number of active vessels will maximize the suppled power per vessel, and reducing the feed pressure will maximize the feed flow rate per vessel. Hence, even minimal wind power can be leveraged to produce fresh water. However, if wind power is zero, the control system cannot help. Even increasing the number of turbines will have no effect. The only available solution is to withdraw water from storage tanks if it exists. Nevertheless, the presence of an online control system provided better overall water production as the water deficit was found to be 6% compared to 7.1% when no control system was implemented.

**Figure 7:**
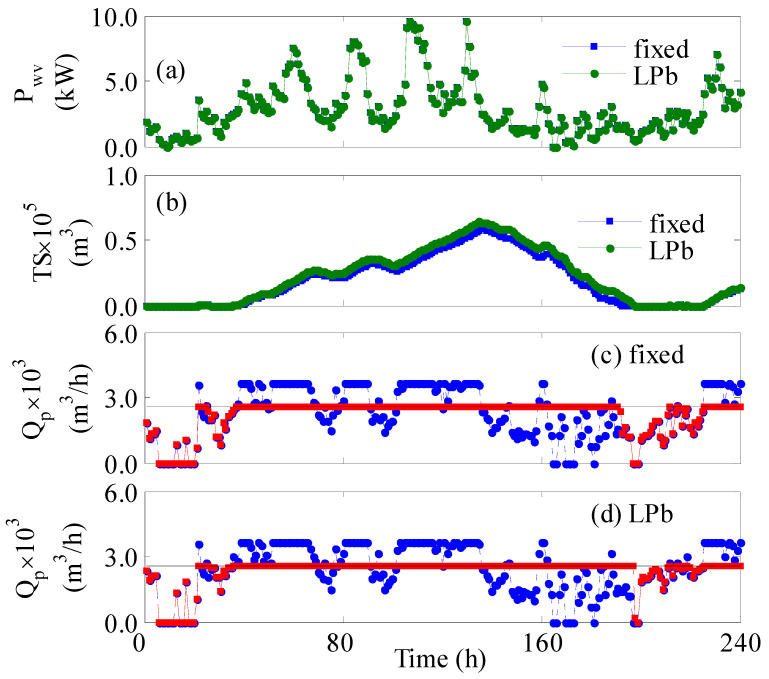
Plant dynamic response for 10 days under a shortage of turbines and vessels; (**a**,**b**) square marker: fixed, circle marker: *LPb*; (**c**,**d**) solid line: $Q_{p}^{s}$; blue circle: $Q_{p}$; red square: $Q_{p}^{r}$.

When the plant operated with a shortage in turbines and vessels, the performance deteriorated as demonstrated in Figure 7. Comparing Figure 7a with Figure 6a, we notice that the supplied power per vessels slightly increased despite the reduction in the number of active turbines. Operating with 80% of the available turbines will reduce the total generated power by 20%; however, the reduction of the active number of vessels to 500 will again increase the power supplied per vessel. The net effect is a slight increase in P_wv_. Nevertheless, the reduction of the active vessels will decrease the total production rate and hence the amount of stored water. The latter is evident when comparing Figure 7b with Figure 6b. This situation led to a water deficit, for both controlled and uncontrolled cases (Figure 7c,d), when the wind power was low during the operation time of 150 to 220 h. This situation is indispensable because the storage tanks were completely depleted as shown in Figure 7b. However, the implementation of online control alleviated the losses by decreasing the total deficit from 14.3% to 9.1%. The superiority of the control system to attenuate the deficit is also obvious graphically in Figure 7c,d, manifested by the profile of the red square, i.e., $Q_{p}^{r}$. 

The above assessment of the process performance was repeated but for full operation over one year. The assessment includes the nominal case, disturbed case, and limited case, i.e., when a partial number of turbines and vessels are enabled. The results are tabulated in Table 3. It is clear that the plant performance in terms of satisfaction of the production in the supply pipelines was not affected by the disturbance for both the controlled and uncontrolled systems. A minimum deficit existed for both cases, which amounted to 0.16~0.2% but at slightly higher product salinity. As mentioned earlier, this inevitable deficit was due to starting the simulation with empty storage tanks. This situation can be eliminated in continuous operation. Notably, the controlled plant exhibited a 20% improvement in the satisfaction of the desired hourly water supply vbyia reducing *LPTHbR* from 0.002 to 0.0016. However, the employed disturbance influenced plant performance in terms of permeate purity. The water purity increased by 24 and 30% in the uncontrolled and controlled systems, respectively. Nevertheless, the obtained water purity was still acceptable since it was less than 0.5 kg/m^3^. On the other hand, when the plant suffered from a shortage in processing units (vessels and turbines), the annual loss of production in the pipelines increased to 0.67% for the uncontrolled system and 0.18% for the controlled system. The controlled system continued outperforming the fixed system by reducing the annual water deficit by 73%. 

It should be noted that all of the above results are based on the proposition of a perfect model for the RO process. Usually, such model-based controllers are tested for robustness, i.e., the ability of the control system to perform as well in the presence of a model–plant mismatch. In the control theory field, there are several tools to deal with such situations such as state and/or parameter estimation technologies. Nevertheless, as mentioned earlier, this is not the scope of this work. The aim here is to investigate the feasibility of the control system, in the best-case scenario, to contribute positively to the performance of the RO desalination plant driven by fluctuating wind power.

## 6. Conclusions

The smooth operation of an RO desalination plant driven by intermittent wind power is a challenging task. The situation is even more difficult when other types of disturbances and/or operational limitations exist. Incorporation of a control system to continuously adapt the plant operation online is necessary to maximize the water production such that the plant can withstand any sudden losses due to intermittent power, partial shutdown for maintenance, and/or operational disturbances. The controlled plant outperformed the one without an online control system by reducing the annual water deficit by 20% under the nominal conditions and when the plant was under disturbances. Furthermore, the control system managed to reduce the annual deficit by 73% when the plant operated partially, i.e., 20% of the turbines and 28% of the vessels were out of service. Using the redistributed hourly water production as the controlled variable (*LPb*) was more effective than using the raw hourly production rate (*LP*). *LPb* creates conservative control actions that do not cause excessive variation that may produce poor water quality. Conversely, *LP* causes excessive control actions because it attempts to maximize the raw production rate as much as possible, sacrificing the permeate purity. However, in addition to measuring the hourly production rate, *LPb* needs an additional measurement of the amount of water stored. 

## Figures and Tables

**Table 1:** Results of the optimal control.

Parameter	Fixed	*LPTR*	*LPTHR*	*LPTHbR*
*N_WT_*	100	100	100	100
*LPTR*	−0.277	−0.379	0.0347	−0.117
*LPTHR*	0.0744	0.1295	0.0404	0.0649
*LPTHbR*	0.0573	0.0652	0.0399	0
*Cp_ave_*	0.322	0.320	0.334	0.305

**Table 2:** Results of the instantaneous control for one-day operation.

Objective	Nominal				Disturbed				
	*LP*		*LPb*		*LP*		*LPb*		Fixed
Option	Free *C_p_*	Bounded *C_p_*	Free *C_p_*	Bounded *C_p_*	Free *C_p_*	Bounded *C_p_*	Free *C_p_*	Bounded *C_p_*	Free *C_p_*
*N_WT_*	100	100	100	100	100	100	100	100	100
*LPTR*	−0.433	−0.484	−0.347	−0.337	−0.451	−0.491	−0.351	−0.351	−0.251
*LPTHR*	0.021	0.021	0.019	0.029	0.015	0.014	0.017	0.017	0.083
*LPTHbR*	0	0	0	0	0	0	0	0	0.070
*Cp_ave_*	0.485	0.414	0.363	0.363	0.598	0.468	0.381	0.381	0.325

**Table 3:** Results of the instantaneous control for a one-year operation.

Case	Nominal		Disturbed		Limited	
Option	Fixed	*LPb*	Fixed	*LPb*	Fixed	*LPb*
*N_WT_*	100	100	100	100	80	80
*LPTR*	−0.338	−0.042	−0.326	−0.020	−0.006	0.012
*LPTHR*	0.147	0.325	0.154	0.232	0.193	0.215
*LPTHbR*	0.0020	0.0016	0.0020	0.0016	0.0067	0.0018
*Cp_ave_*	0.327	0.305	0.406	0.406	0.328	0.352

## Data Availability

Not applicable.

## Nomenclature

*A*	membrane permeability, m/h.bar
*A_s_*	membrane surface area, m^2^
*B*	membrane solute permeability, m/h
*b_π_*	osmotic coefficient, m^3^·bar/kg
*C_f_*, *C_p_*, *C_c_*	salt concentration in feed, permeate and brine, kg/m^3^
*C_b_*, *C_m_*	average salt concentration and salt concentration at membrane wall, kg/m^3^
$C_{pave}$	Average permeate salinity, kg/m^3^
*J_w_*	water flux, m/h
*k_s_*	mass transfer coefficient, m/h
*LP*	instantaneous loss of production, m^3^/h
*LPb*	instantaneous loss of redistributed production, m^3^/h
*LPTHT*	total loss of hourly production ratio
*LPTR*	total loss of total production ratio
*LPTHbR*	total loss of hourly redistributed production ratio
*N_WT_*	number of wind turbines
*Nv*	global number of vessels
*n_e_*	number of RO elements in a vessel
*n_l_*	number of leaves per RO element
*P_w_*	generated wind power per turbine, W
*P_wv_*	supplied wind power per vessel, W
*P_f_*, *P_b_*	feed, permeate, and brine pressure, bar
*P_drop_*	pressure drop, bar
*Q_c_*	brine volumetric flow rate per vessel, m^3^/h
*Q_b_*	mean volumetric flow rate through membrane channel, m^3^/h
*Q_fv_*, *Q_pv_*	feed and permeate flow rate per vessel, m^3^/h
$Q_{p}^{s}$	water demand of the city, m^3^/h
*Q_p_*	Total water production, m^3^/h
$Q_{p}^{r}$	Redistributed production rate, m^3^/h
*R_c_*	recovery ratio (%)
*ST*	water storage tank capacity, m^3^
*ST^m^*	maximum stored water, m^3^
*t_k_*	time at step k, h
*Greek*	
*η_p_*	pump efficiency
*π*	osmotic pressure, bar

## Appendix A. RO Model

(A1)
$$Q_{fv}=\frac{3600\times P_{wv}}{P_{f}\times 1\times 10^{5}\eta_{p}^{-1}}$$

(A2)
$$Q_{pv}=Q_{fv}R_{c}$$

(A3)
$$Q_{fv}=Q_{pv}+Q_{cv}$$

(A4)
$$Q_{fv}C_{f}=Q_{pv}C_{p}+Q_{cv}C_{c}$$

(A5)
$$C_{b}=\frac{C_{f}+C_{c}}{2}$$

(A6)
$$J_{w}=A(\Delta P-\Delta \pi );\;\Delta P=P_{f}-P_{b}-P_{drop/2};\;\Delta \pi =b_{\pi }(C_{m}-C_{p})$$

(A7)
$$P_{drop}=9.5\times 10^{8}{\left(\frac{Q_{f}+Q_{c}}{2\times 3600}\right)}^{1.7}$$

(A8)
$$J_{w}=A\left[\Delta P-b_{\pi }\left(\frac{BC_{b}\exp\left(J_{w}/k_{s}\right)}{J_{w}+B\exp\left(J_{w}/k_{s}\right)}\right)\exp\left(J_{w}/k_{s}\right)\right]$$

(A9)
$$C_{p}=\frac{BC_{p}}{B+J_{w}\exp(\frac{J_{w}}{k_{s}})}$$

(A10)
$$C_{m}=C_{p}+\left(C_{b}-C_{p}\right)\exp\left(j_{w}/k_{s}\right)$$

(A11)
$$Q_{p}=J_{w}A_{s}n_{e}n_{l}N_{v}$$
